# Expanding treatment frontiers in hidradenitis suppurativa: Double immunomodulator therapy in severe refractory cases

**DOI:** 10.1016/j.jdcr.2026.08.026

**Published:** 2026-08-23

**Authors:** Christina Tolete, Adam Friedman, Teresa Duong, Leonardo Tjahjono

**Affiliations:** a Department of Dermatology, School of Medicine and Health Sciences, George Washington University, Washington, District of Columbia; b Sidney Kimmel Medical College, Thomas Jefferson University, Philadelphia, Pennsylvania

**Keywords:** Chronic inflammatory skin disease, Dual biologic therapy, Hidradenitis suppurativa, HiSCR (Hidradenitis Suppurativa Clinical Response), Immunomodulators

## Introduction

Hidradenitis suppurativa (HS) is a debilitating, chronic inflammatory skin condition that affects quality of life (QOL) significantly.^1^ While there are 3 EMA and FDA-approved medications, there is still significant unmet need for patients with HS. Most available and upcoming biologics and small molecule inhibitors (SMI) that are still in clinical trials use HS Clinical Response (HiSCR-50) and International Hidradenitis Suppurativa Severity Score System 55% Response (International Hidradenitis Suppurativa Severity Score System [IHS4]-55) to monitor response, which is only 50% to 55% improvement in HS.^2^ While psoriasis (PsO) and atopic dermatitis (AD) have treatment options that can reliably achieve 75% to 90% improvement for severe disease, this is a far cry for HS, signifying the disease burden and an unmet need in controlling the disease activity.

This case series includes adult patients with severe refractory HS, which we classified as HS patients with minimum debilitating pain level of 6 and IHS4 score above 10 despite trials of many multiple monotherapy biologics and SMI with combinations of adjunctive treatments, that ultimately utilized a combination of biologics and/or SMI. Dual biologics and/or SMI were utilized due to failure of aforementioned combination regimen failure in achieving reasonable HS disease activity and pain control. The study was approved by the George Washington University institutional review board department.

## Case presentation

Included cohorts are severe refractory HS patients that have trialed monotherapy biologic or SMI with various combinations of other non-immunomodulatory treatments, including topical and systemic antibiotics, hormonal, and weight-loss modulators (Table I). Median IHS4 level prior to initiation of any biologic or SMI was 25 (range 17-31) and median pain level of 9 (range 6-10), signifying the high disease burden of the cohort. While there was modest improvement to median IHS4 score of 20 (range of 14-25) after trials of multiple monotherapy biologic or SMI in combination with other non-biologic or SMI of 6 months each (Tables I and II), the severe disease burden necessitated additional advanced therapy by combining another biologic and/or SMI and thus these are the patients that are included in this retrospective case series. The authors felt that due to high risk of permanent scarring and formation of further draining tunnels, which can further make the HS harder to treat, pursuing dual advanced therapies to control the active inflammatory HS rapidly is necessary. Most patients tried at least 3 different biologics or SMI before starting dual combination biologics and/or SMI. All therapies listed in Table II represent prior monotherapy biologic or small molecule inhibitor exposures that were trialed in monotherapy fashion before being escalated to dual therapy. The second biologics and/or SMIs were obtained through letter of appeal. Clinical efficacy was monitored with the IHS4 metric and pain level, which are the preferred metric used due to continuous disease monitoring. Our clinic utilizes IHS4 rather than HiSCR scoring to measure a more accurate and real time disease activity and burden. The score precisely monitors the depth and significance of treatment outcome regimen, while still accounting for draining tunnel activities, which the majority of our severe HS patients exhibit.

**Table I:** Baseline characteristics, prior therapies, and dual targeted treatment regimens in patients with refractory hidradenitis suppurativa

Characteristic	No (%)
Total patients	9
Female	6 (66)
Black	8 (89)
Anatomical sites involved	
>2	6 (66)
2	2 (22)
1	1 (11)
Comorbidities	
Obesity	6 (66)
Hyperlipidemia	5 (55)
Type II DM	4 (44)
PCOS	3 (33)
Crohn's	3 (33)
AsP	2 (22)
Psoriatic arthritis	1 (11)
Multiple sclerosis	1 (11)
Psoriasis	1 (11)
Concomitant systemic	
Spironolactone	6 (66)
Doxycycline	5 (55)
Glp-1 agonist	3 (33)
Oral Metformin	2 (22)
Oral Roflumilast	2 (22)
Dutasteride	1 (11)
Previous monotherapy biologics or small molecule inhibitors use	
Adalimumab	8 (89)
Infliximab	8 (89)
Secukinumab	5 (55)
Bimekizumab	5 (55)
Upadacitinib	3 (33)
Risankizumab	3 (33)
Guselkumab	3 (33)
Anakinra	1 (11)
Combinations to achieve desirable IHS4	
Bimekizumab + Upadacitinib	4 (44)
Infliximab + Upadacitinib	3 (33)
Secukinumab + Upadacitinib	1 (11)
Adalimumab + Secukinumab	1 (11)

Longitudinal changes in IHS4 and pain level		
	IHS4 [median, (range)]	Pain level [median, (range)]
Prebiologic initiation	25 (17-31)	9 (6-10)
6 mo post monotherapy initiation	20 (14-25)	8 (6-10)
4 wk post double biologics initiation	16 (9-19)	7 (5-9)
16 wk post double biologics therapy	7 (2-9)	4 (1-6)

*AsP*, Ankylosing spondyloarthropathy, *DM*, diabetes mellitus; *GLP-1*, glucagon-like peptide-1; *IHS4*, International Hidradenitis Suppurativa Severity Score System; *No*, Number; *PCOS,* polycystic ovary syndrome.

**Table II:** Patient-level characteristics, prior monotherapy biologic or small molecule inhibitor exposures, and clinical outcomes following dual therapy

Gender, age, race	Previous monotherapy biologics or small molecule inhibitors used	Comorbidities	Final Dual therapy	Areas involved	IHS4 (Pre-biologic → 6-mo post-monotherapy initiation → 16-wk post-dual therapy)	Pain level (Pre-biologic → 16-wk post-dual biologic)	Note
F,18,B	ADA, IFX	Obesity, HLD, T2DM	BKZ, UpA	Axillae	28, 25, 8	9, 3	Developed URI that did not recur or result in discontinuation of therapy when she was on dual therapy period
F,23,B	ADA, IFX, SEC, BKZ	PCOS, AsP, HLD	BKZ, UpA	Inner thighs, inguinal folds	30, 24, 8	8, 6	
F, 27,B	ADA, IFX, UpA, RZK, GUS	Crohn's, Obesity, T2DM	IFX, UpA	Axillae, inner thighs	17, 14, 2	6, 1	IHS4 increased at the end of 4 mo cycle with risankizumab and gulsekumab, but Crohn's disease remained under great control
F, 33, B	SEC, BKZ	MS, Obesity	SEC, UpA	Buttocks	19, 15, 7	7, 5	
F, 34, B	ADA, IFX, UpA, RZK, GUS	Crohn's, AsP, Obesity	IFX, UpA	Axilla, inframammary breasts	25, 20, 7	9, 4	IHS4 increased at the end of 4 mo cycle with risankizumab and gulsekumab
F,47,B	ADA, IFX, SEC, BKZ	PsO, PsA	SEC, UpA	Buttocks, inguinal folds	27, 22, 3	9, 3	The patient had severe oral candidiasis while she was on bimekizumab and upadacitinib combination, while did not develop any on bimekizumab monotherapy. Psoriasis achieved durable complete clearance 6 mo post dual therapy
M, 22, B	ADA, IFX, SEC, BKZ, ANA	Obesity, HLD, T2DM	BKZ, UpA	Axillae, buttocks	24, 17, 4	7, 2	Developed URI that did not recur or result in discontinuation of therapy when she was on dual therapy period
M, 33, B	ADA, IFX, UpA, RZK, GUS	Crohn's, PCOS, hyperlipidemia, T2DM	IFX, UpA	Inframamary and intermammary breasts	24, 19, 6	10, 5	
M, 38, W	ADA, IFX, SEC, BKZ	PCOS, Obesity, HLD	BKZ, UpA	Axillae, inner thighs	31, 23, 9	10, 6	Developed URI that did not recur or result in discontinuation of therapy when she was on dual therapy period

*ADA*, Adalimumab; *ANA*, Anakinra; *AsP*, Ankylosing spondyloarthropathy; *B*, Black; *BKZ*, Bimekizumab; *F*, Female; *GUS*, Guselkumab; *HLD*, Hyperlipidemia; *IFX*, Infliximab; *IHS4*, International Hidradenitis Suppurativa Severity Score System; *M*, Male; *MS*, Multiple Sclerosis; *PCOS*, Polycystic ovary syndrome; *PsA*, Psoriatic Arthritis; *PsO*, Psoriasis; *RZK*, Risankizumab; *SEC*, Secukinumab; *T2DM*, Type II Diabetes Mellitus; *UpA*, Upadacitinib; *URI*, Upper Respiratory Infection; *W*, White.

**Fig 1:**
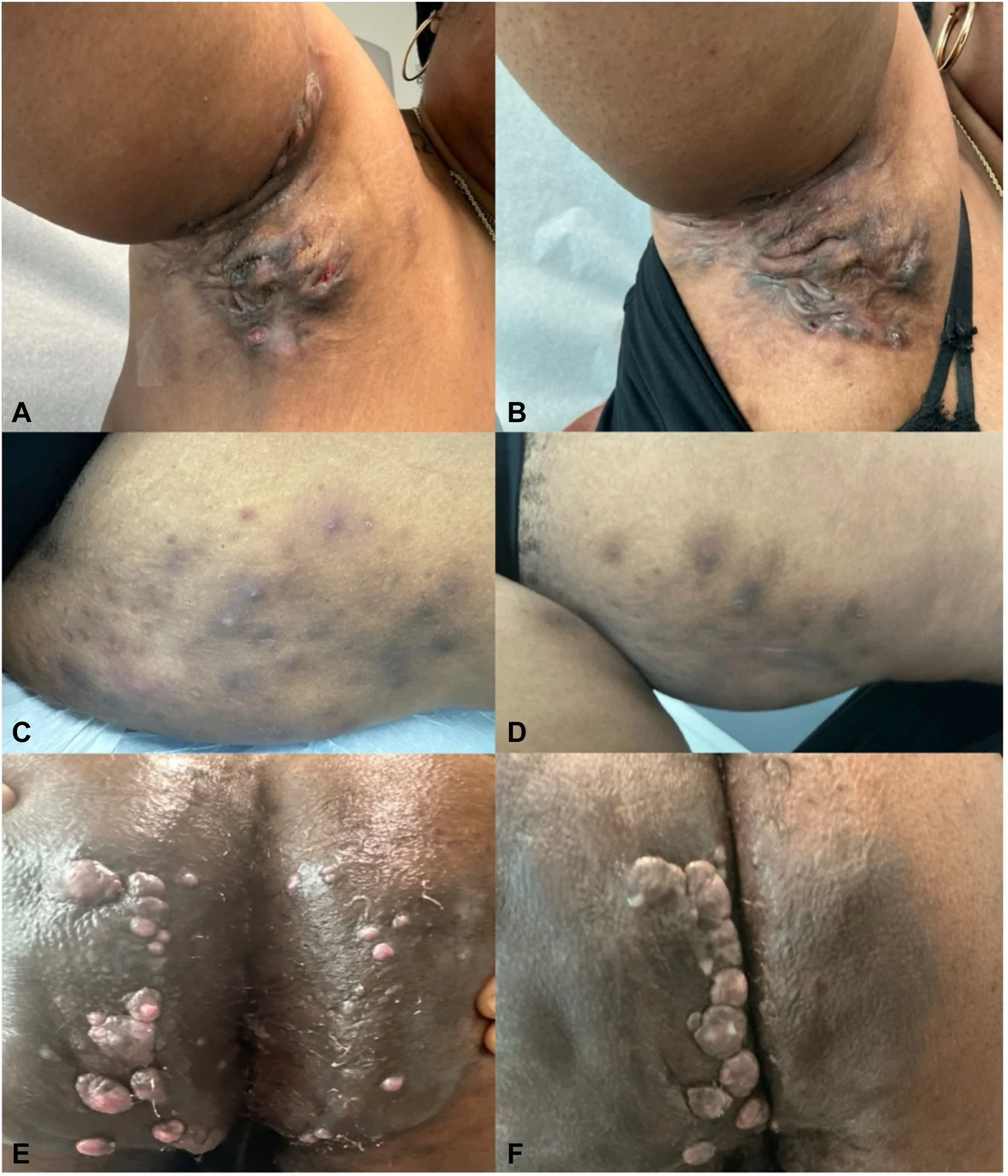
Selected clinical photographs demonstrating improvement of severe hidradenitis suppurativa following dual biologic therapy. **A, C,** and **E,** Baseline images demonstrate numerous inflammatory papules, pustules, and nodules, several of which are ulcerated, with surrounding erythema, scarring, and post-inflammatory hyperpigmentation. **B, D,** and **F,** After initiation of dual biologic therapy (upadacitinib + bimekizumab in **A–B**; infliximab + upadacitinib in **C–D**; upadacitinib + secukinumab in **E–F**), there is marked clinical improvement characterized by reduction in inflammatory nodules, flattening of lesions, and resolution of active inflammation, leaving residual scarring and post-inflammatory hyperpigmentation.

Of the 9 patients included, 6 (66%) were female. Eight patients (89%) were Black. Eight patients (89%) had prior exposure to adalimumab and infliximab, and 5 (55%) with secukinumab and bimekizumab. The most common non-immunomodulators systemic therapies used alongside double immunomodulator regimen was spironolactone (6, 66%). Three patients (33%) had recalcitrant Crohn’s, 2 (22%) had recalcitrant ankylosing spondyloarthropathy, and 1 (11%) had recalcitrant psoriatic arthritis, which all also improved only after initiation of double immunomodulators (Tables I and II). Prior biologic therapies included maintenance of 40 mg weekly adalimumab, 300 mg secukinumab every 2 weeks, bimekizumab 320 mg every 4 weeks, and infliximab 10 mg/kg every 4 weeks after their loading dose. Three patients with comorbidities of Crohn’s disease obtained FDA-approved Crohn’s dosing of upadacitinib (45 mg oral once daily for 12 weeks, then 30 mg once daily during monotherapy period, however we elected to use the 15 mg once daily dosing during the dual therapy period when combined with a biologic), Risankizumab (intravenous 600 mg at weeks 0, 4, 8, then subcutaneous 300 mg every 8 weeks thereafter) and guselkumab (subcutaneous 200 mg week 0,4,8, then 100 mg every 8 weeks thereafter). The most common final immunomodulatory combination was bimekizumab and upadacitinib (4, 44%). One patient developed oral candidiasis while on combination of bimekizumab and upadacitinib therapy, which the bimekizumab was switched to secukinumab; the oral candidiasis responded well with a course oral fluconazole and did not recur. All patients were able to continue the double immunomodulators for at least 6 months, with no safety signal. We monitored bloodwork every 3 months as a standard in our HS referral clinic bloodwork that include complete blood count with differential, complete metabolic panel, creatine kinase, and lipid panel, the latter 2 if the patient is receiving upadacitinib. No severe infections, including herpes zoster, were observed despite no vaccination recommendations and routine laboratory monitoring did not reveal any concerning abnormalities in our cohort. Special attention was given on patients receiving upadacitinib and with comorbidities of hyperlipidemia and hypercholesterolemia; there was no safety signal noted. Three patients developed upper respiratory infection in those receiving upadacitinib 15 mg and bimekizumab 320 mg every 4 weeks during the dual therapy period, but self-resolved and did not result in discontinuation of therapy. Median IHS4 improved by 18 and 5 for pain level from initiation of any biologic to 16 weeks post initiation of double biologic (Tables I and II). Clinical photographs demonstrating treatment response in three patients are shown in Fig 1.

## Discussion

We report the largest cohort of severe recalcitrant HS management with dual immunomodulators regimen. While AD and PsO have reliable treatment options, double immunomodulators sometimes are necessary.^3^ Literature on this regimen is very sparse with only 3 reported cases.^4^ Despite a bias from the authors’ experience as an HS referral center that evaluates severe HS disease, the reported HiSCR-50 and IHS55 in phase 3 pivotal trials for both FDA and EMA-approved therapies are relatively low for monotherapy treatments. This mirrors our experience, necessitating the treatment regimen represented in this case series.^2^

A key strength of this study is the inclusion of a predominantly Black patient population with a high burden of socioeconomic disadvantage, a group that is disproportionately affected by hidradenitis suppurativa yet remains markedly underrepresented in biologic and combination-therapy studies. By capturing real-world outcomes in this population, our findings enhance the generalizability of dual-biologic treatment data and provide clinically relevant insights for populations most impacted by severe and refractory disease.

Dual biologic regimen has been utilized in other chronic inflammatory conditions, such as Crohn’s disease, which we observed in our cohorts with recalcitrant inflammatory comorbidities that only improved after initiation of dual biologics, highlighting the broad applicability.^5^ While our patients tolerated the regimens well, the theoretical immunosuppressive effects can be of concern. Additionally, not every patient may be suitable with the combination we reported given theoretical black box warning of upadacitinib such as major adverse cardiovascular events, thrombosis, and malignancy. Fortunately, none of our patient has risk factors that increases risk to such adverse events. IHS4 is preferred in our study over HiSCR because it provides a continuous measure of active disease burden that captures lesion severity and partial response—particularly in tunnel-predominant and severe HS—whereas HiSCR is a binary endpoint that underestimates meaningful improvement.

Although inflammation in HS is often classified as a disorder of T-helper 17 immune axis, which include the linear cascade of TNF-α and IL-17 upregulation, which is further supported from clinical response to available treatment agents such as TNF-α and IL-17 inhibitors, the disease is more multifaceted and incredibly complex. HS also involves innate and adaptive immune dysregulation, with complex interactions of heterogeneous cytokines, T cells, B cells, neutrophils that have yet to be addressed by FDA-approved therapies.^6^ We combined upadacitinib, a JAK-1 inhibitor, with TNF-α and IL-17A/F inhibitors, because the biologics only target one pathway and upadacitinib modulate unaddressed, but potentially involved, multiple downstream effector pathways simultaneously, such as IL-1, IL-6, IL-36 and interferon-γ.^6^ Approved therapies do not modulate all immune dysregulation. Due to the diverse inflammatory milieu, immunomodulation of one cytokine likely would result in incomplete response in certain patients. Looking ahead, the same principle of multi-pathway modulation is being harnessed through bispecific antibody platforms. Emerging agents such as brivekimig, a TNF/OX40L nanobody, and NM26, an IL-4/IL-31bispecific antibody, exemplify this next generation of “built-in combination” biologics designed to simultaneously target distinct inflammatory axes. Therapies with different mechanistic targets are in development, thus providing novel targets should double biologics become more acceptable in the future. However, conclusions should be interpreted cautiously given the limitation of case series. Randomized controlled studies are needed to assess the efficacy and safety of the treatment regimen, especially given the unknown depth immunosuppressive property of these combinations and the practice of utilizing dual biologic and/or SMI is not yet widely adopted.

## Conflict of interest

Dr Leonardo Tjahjono has served as a consultant and/or speaker for Arcutis, Bristol Myers Squibb, Eli Lilly, Incyte, Leo pharma, UCB, J&J, Regeneron, Disc medicine, Novartis, Sun Pharma, Curio Science and Galderma. Dr Adam Friedman consults/Ad board: La Roche Posay, Galderma, Kenvue, Microcures, Leo Pharma, Pfizer, Hoth Therapeutics, Zylo Therapeutics, Mino Labs, J&J, Arcutis, Lilly, UCB, Novartis, Regeneron/Sanofi, Takeda; Speaker: Regeneron/Sanofi, J&J, Incyte, UCB, Galderma, Arcutis, Lilly, Pfizer, Novartis; Grants: Pfizer, Lilly, Galderma, Incyte, J&J, Abbvie, Loreal, Regeneron/Sanofi. Christina Tolete and Teresa Duong do not have any disclosures.
